# ALA Promotes Sucrose Accumulation in Early Peach Fruit by Regulating SPS Activity

**DOI:** 10.3390/cimb46080469

**Published:** 2024-07-24

**Authors:** Zheng Chen, Xin Guo, Jinhua Du, Mingliang Yu

**Affiliations:** 1 Institute of Pomology, Jiangsu Academy of Agricultural Sciences, Jiangsu Key Laboratory of Horticultural Crop Genetic Improvement, 50 Zhongling Street, Nanjing 210014, China; 20230072@jaas.ac.cn (Z.C.);; 2 College of Horticulture, Nanjing Agricultural University, Nanjing 210095, China

**Keywords:** 5-aminolevulinic acid, peach, SPS activity, PpSPS2

## Abstract

5-Aminolevulinic acid (ALA), as a novel plant growth regulator, is a critical precursor for the biosynthesis of porphyrin compounds in all organisms. Many studies have reported that exogenous ALA treatment could improve fruit sweetness. However, the mechanism by which ALA promotes the increase in sugar content in fruit remains unclear. In this study, we found that ALA significantly promoted sucrose accumulation and SPS (sucrose phosphate synthase) activity in peach fruit. At 14, 28, 42, 50 and 60 days after ALA treatment, sucrose content of fruit was increased by 23%, 43%, 37%, 40% and 16%, respectively, compared with control treatment, and SPS enzyme activity was increased by 21%, 28%, 47%, 37% and 29%, respectively. Correlation analysis showed that the sucrose content of peach fruit under ALA treatment was significantly positively correlated with SPS activity. Subsequently, bioinformatics was used to identify SPS gene family members in peach fruit, and it was found that there were four members of the *PpSPS* gene family, distributed on chromosomes 1, 7 and 8, named *PpSPS1*, *PpSPS2*, *PpSPS3* and *PpSPS4,* respectively. The results of qRT-PCR showed that *PpSPS2* and *PpSPS3* were highly expressed in response to ALA during fruit development, and the expression of *PpSPS2* was positively correlated with SPS activity and sucrose accumulation in peach fruit. The results of tobacco subcellular localization showed that PpSPS2 was mainly distributed in the cytoplasm and nucleus, while PpSPS3 was mainly distributed in the nucleus. The results of this study will lay the foundation for further study on the functions of *PpSPS* and the regulation of sugar metabolism during the development and ripening of peach fruit by ALA.

## 1. Introduction

The peach (*Prunus persica* L.) is a small deciduous tree belonging to the peach subgenus of the rose family, and its fruits are popular in domestic and foreign markets. Sugar is one of the important factors in determining the flavor of peach fruit, and the sweet taste of peach fruit is often more favored by consumers. At present, China’s peach cultivation area and output are ranked first in the world. Early, middle and late varieties of planting, enrich the fruit market, which also brings huge income to growers [1]. However, most early-maturing varieties have weak fruit flavor and low quality [2]. The sugar accumulation in peach fruit is determined by the metabolism of photosynthetic products from “source” to “sink” loading, transportation, unloading and carbohydrate transformation in the fruit [3]. The medium- and late-maturing varieties have a longer fruity period and a longer photosynthetic time, so the accumulated soluble sugar content is relatively high. Therefore, efforts to improve the quality of early peaches have become one of the important goals of peach production in China.

In ripe peach fruit, the main sugar is sucrose, followed by reducing sugar, including glucose and fructose, and the sorbitol content is relatively low [4]. Although sorbitol is the main transport form of photosynthetic products in *roseaceae*, it still plays an important role in sucrose metabolism in fruit [5]. The main enzymes involved in sucrose metabolism in fruit are sucrose invertase, sucrose synthase (sucrose synthase, EC 2.4.1.13, SuS) and sucrose phosphate synthase (SPS, EC 2.4.1.14) [6]. SuS and SPS were related to sucrose synthesis and catalyzed the reversible reaction of uridine diphosphate glucose (UDGP) and fructose-6-phosphate with UDGP and fructose to sucrose production, respectively [7,8]. The changes in sucrose metabolizing enzyme activity in fruit were closely related to the level of sugar accumulation and the composition of fruit sugar [9]. Exogenous hormones, external environmental factors and cultivation measures also have important effects on the sugar content of fruits. It was found that the use of exogenous ABA and GA_4+7_ at a certain concentration could effectively increase the accumulation of sugar in peach fruit. Moderate water stress can increase the sugar content of fruit, induce an increase in ABA levels in peach fruit and activate sorbitol metabolism, thus promoting sugar accumulation [10,11]. Kumar et al. (2010) pruned peaches to three different degrees: light, medium and heavy. The results showed that moderate and severe pruning could significantly improve the quality, including soluble sugar content [12].

5-Aminolevulinic acid (ALA), as a popular growth regulator, is a key precursor for the biosynthesis of porphyrin compounds in all organisms [13]. It can increase the net photosynthetic rate of leaves, enhance plant stress resistance, promote plant growth, improve yield and quality when promoting chlorophyll synthesis, and have a broad application prospect in agriculture and forestry production [14,15,16]. In terms of improving fruit quality, ALA not only promoted anthocyanin accumulation in apples and other colored fruits, but also promoted sugar accumulation in fruits and reduced titratable acid content [17]. Recent research also showed that ALA improved internal qualities, such as soluble protein content and enhanced fruit flavor and nutrient content [1].

However, the mechanism by which ALA promotes the accumulation of sugar in fruit is still lacking. The results of the determination of sucrose showed that ALA could significantly promote sucrose accumulation in peach fruit. The qRT-PCR results showed that *PpSPS2* expression was significantly positively correlated with sucrose content in peach fruit, and ALA could significantly promote SPS activity, suggesting that ALA played an important role in promoting peach sucrose accumulation. Therefore, the study of peach sucrose metabolism and regulation mechanisms is of great significance for improving fruit quality, commodity value and market competitiveness and has been a hot spot in the field of fruit sugar metabolism research.

## 2. Materials and Methods

### 2.1. Plant Materials and Chemical Treatments

The experiment was conducted from March 2021 to June 2022 in Nanjing, China, with a subtropical monsoon climate, abundant rainfall, and four distinct seasons. The experimental variety was *Prunus persica* ‘Zhongyoutao 4’, which were four-year-old nectarine trees, arranged in east–west rows with a plant spacing of 1 m × 4 m and Y-shaped pruning. ‘Zhongyoutao 4’is an early-mature nectarine variety with yellow flesh, a pleasant aroma, and high quality. Two treatments were set up at different growth and development stages of peach fruit, including the control (spraying with clean water) and the ALA treatment (spraying with 10 mg L^−1^ ALA). The ALA concentration used was referred to by Guo Lei et al. (2013) [18]. The ALA solution was foliar sprayed on 10 April, 20 days after full blooming, when the young fruits were at the cell division stage. An ALA solution of 10 mg L^−1^ was sprayed at the second rapid expansion period of fruit development (23 May, 60 d after full blooming). Eight peach trees with similar growth potential were selected for each treatment and randomly arranged in a single plot. Unified cultivation and management measures were adopted after treatment.

### 2.2. The Determination of Physiological and Biochemical Indicators of Peach Fruits

Determination of individual fruit weight: 20 fruit weights were accurately weighed with a balance and averaged. Determination of soluble solids in fruit: The equatorium-symmetric position on the left and right of the suture line of each fruit was selected, measured by a PAL-1 digital display sugar meter, and the reading was recorded. Each treatment was repeated 20 times, and the average value was taken. The content of total soluble sugar in fruit was determined by the anthrone colorimetric method [19]. The content of total soluble protein in fruit was determined by the Coomassie brilliant blue method [19]. The titrable acid content of the fruit was determined by acid-base titration [20]. The content of vitamin C in fruit was determined by the UV-visible light method [21].

### 2.3. Sucrose Determination

The nectarine fruits of the field experiment were collected after ALA treatment, including 14, 28, 40, 50 and 60 days after treatment. The soluble sugars of nectarine fruits were determined using high-performance liquid chromatography (HPLC). The method of determination of sucrose in peach fruit samples was based on Liang et al. [1]. The chromatographic conditions were as follows: liquid chromatography (Acquity UPLC H-class, Waters, Milford, MA, USA), Prevail carbohydrate ES 5u column (100 mm × 4.6 mm, 5 μm), ELSD detector, column temperature 50 °C, mobile phase: acetonitrile/water = 80/20 (*v*/*v*), injection volume 2 μL, and flow rate 1.0 mL min^−1^. The ELSD2000 evaporative light scattering detector parameters were as follows: nitrogen as the carrier gas, gas flow 1.5 mL min^−1^ and drift tube temperature 80 °C. The content of sucrose in the fruits was calculated according to the sample peak areas and standard curves of each sugar. At least three biological repetitions were conducted.

### 2.4. Determination of SPS Activity

Total protein of peach fruit under different treatments was extracted with the plant total protein extraction kit (Nanjing Kangwei Century Biotechnology Co., Ltd., Nanjing, China, item No. CW0885S), and total protein concentration was determined with the BCA protein quantitative kit (Kangwei Century Biotechnology Co., LTD., item No. CW0014S). The total protein concentration of peach fruit under different treatments was diluted to the same concentration before determining the PpSPS activity of peach fruit. A sucrose phosphate synthetase (SPS) activity assay kit (Shanghai Fusheng Industrial Co., Ltd., Shanghai, China, Item No. A112622) and microcoder were used to detect SPS activity. Three biological replicates were taken and averaged.

### 2.5. Identification of SPS Genes in Peaches and Other Plants

The Hidden Markov Model (HMM) [22] analysis and a Simple Modular Architecture Research Tool (SMART) [23] were used for the search. The HMM profile was downloaded from the Pfam protein family database (http://pfam.xfam.org/) to obtain the Pfam number of the protein sequence. The domain contained in the target protein sequence was detected using the HMM search command in the HMMER package, with an e-value ≤1 × 10^−3^. The results of the HMMER sequence alignment were screened to remove protein sequences that were 45% longer than the length of the HMM model domain while retaining the longest protein sequence in the variable shear. All nonredundant protein sequences were retrieved and further analyzed with SMART (a Simple Modular Architecture Research Tool) (http://smart.embl-heidelberg.de/) to examine the results. The same genes were confirmed as family members. Gene structure analysis: the information about exon-intron structures was acquired from reference genome annotation files. The alignment of the cDNAs with their corresponding genomic DNA sequences. Chromosomal distribution: the location information of the genes and the conserved regions were confirmed by reference genome annotation files and then plotted using the SVG package of the Perl programming language.

### 2.6. Bioinformatics Analysis

The MEME program (http://alternate.meme-suite.org/tools/meme, accessed on 25 July 2023) was used to identify the motifs in the gene family sequences [24]. The maximum motif search value was set at 15 and an optimum motif width of 10-100 amino acid residues. Other parameters are default. The phylogenetic tree was constructed with the neighbor-joining algorithm in MEGA (version 7.0) [25] with a bootstrapping test of 1000 times. The gene structure and motifs were analyzed using a systematic evolution relationship.

### 2.7. RNA Isolation and Quantitative Real-Time Fluorescence PCR (qRT-PCR)

After fruit samples were frozen in liquid nitrogen, use kits (Cat. No. 19291ES50; Cat. No. 11121ES60, Yeasen Biotechnology (Shanghai, China) Co., Ltd.) to extract and reverse transcription, respectively, following the manufacturer protocols. Design primers for real-time PCR using Primer 5 software, with *PpTEF* (translation elongation factor) gene as the internal reference gene. The RT-qPCR was performed as previously described [1]. The primer sequences used are listed in Table S1. The 2^−ΔΔCT^ method was used to calculate the relative expressions of genes [26]. Three independent biological replicates were performed for each experiment.

### 2.8. Subcellular Localization of PpSPS2 and PpSPS3 in N. benthamiana

The WoLF PSORT (http://www.genscript.com/wolf-psort.html, accessed on 25 July 2023) was performed to predict the subcellular localization of *PpSPS2* and *PpSPS3*. The *PpSPS2* and *PpSPS3* without the stop codon removed were cloned and constructed into the pCambia1302 vector containing green fluorescent protein (GFP) tag to obtain the recombinant plasmids pcambia1302-*PpSPS2*-GFP and pCambia1302-*PpSPS3*-GFP, which were then transformed into *GV3101* competent cells (Shanghai Weidi Biotechnology Co., Ltd., Shanghai, China, CAT#: AC1001). The determination of the subcellular location of *PpSPS2* and *PpSPS3* was performed as previously described [27]. The primer sequences used are listed in Table S1.

### 2.9. Statistics and Reproducibility

The data are the means of at least three independent biological replications. Statistical analyses are performed using a two-sided Student’s *t*-test or a one-way ANOVA followed by mean separation with Tukey’s honestly significant difference test or Duncan’s multiple range test.

## 3. Results

### 3.1. ALA Promoted the Sucrose Content and SPS Activity of Peach Fruit during Fruit Development

**Figure 1:**
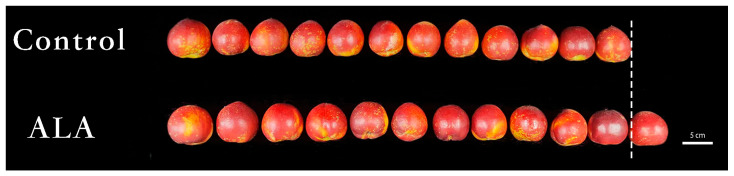
Effects of exogenous ALA treatment at different stages of fruit development on the apparent quality of ‘Zhongyoutao 4’ nectarine ripe fruit (12 fruits per treatment are shown here).

Comparing the same number of peach fruits in different treatment groups under the same sampling standard, the results showed that the peach fruit in the ALA treatment group was significantly larger than that in the control group (Figure 1), and the average fruit weight was significantly higher than that in the control group (Table 1), suggesting that spraying ALA at the fruit expansion stage could promote fruit growth. In addition, ALA treatment could significantly increase the contents of soluble solids, soluble sugar and soluble protein in peach fruit (Table 1).

### 3.2. ALA Promoted the Sucrose Content and SPS Activity of Peach Fruit during Fruit Development

**Figure 2:**
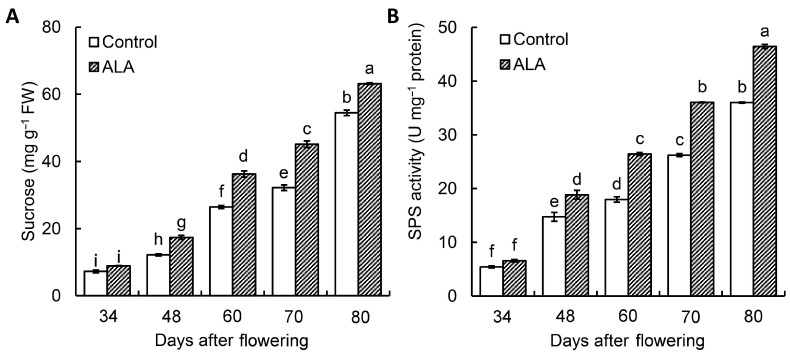
The effects of ALA on sucrose content (**A**) and SPS activity (**B**) of peach fruit during fruit development. The values are the means ± SE from three biological replicates. The same letters above the bars in each enzyme indicate no significant differences at *p* = 0.05 level.

After 48 days of flowering ALA treatment, we found that the sucrose of the peach was significantly improved (Figure 2A). In addition, we also found that ALA significantly improved the SPS activity of the peach fruits (Figure 2B). Correlation analysis showed that sucrose content in peach fruit was positively correlated with SPS activity (Table S2 and Figure S1).

### 3.3. Identification and Characterization of the PpSPS Gene Family

**Figure 3:**
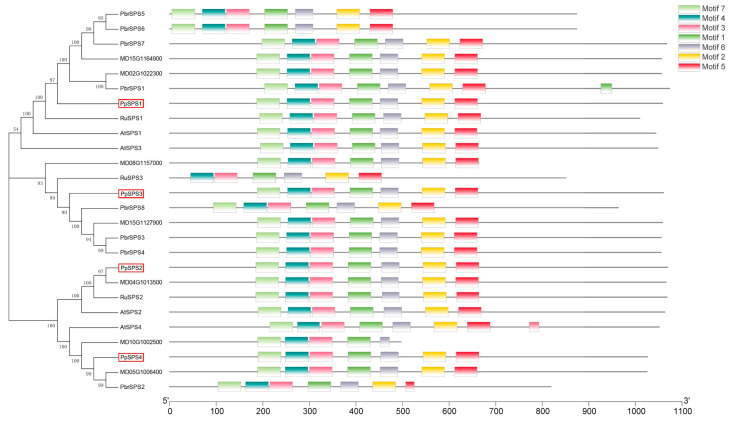
Bioinformatics analysis of SPS. The red boxes are identified members of the PpSPS gene family.

In order to further study how ALA regulates PpSPS activity, we conducted a bioinformatics analysis of *PpSPS*. The results showed that there were four members of the *PpSPS* gene family, namely *PpSPS1*, *PpSPS2*, *PpSPS3* and *PpSPS4* (Table 2), and all genes had seven acting elements (Figure 3).

### 3.4. Chromosomal Location, Gene Structure and Promoter Analysis of PpSPS Family Genes

**Figure 4:**
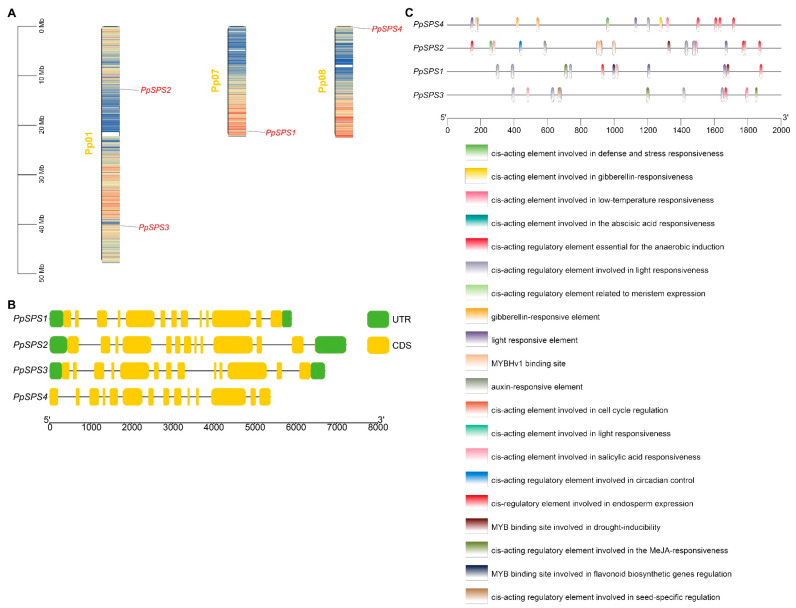
Chromosomal location (**A**), gene structure (**B**) and promoter analysis (**C**) of *PpSPS* family genes.

Chromosome localization analysis showed that *PpSPS2* and *PpSPS3* were distributed on chromosome 1, *PpSPS1* was distributed on chromosome 7; and *PpSPS4* was distributed on chromosome 8 (Figure 4A). The results of gene structure analysis showed that *PpSPS1*, *PpSPS2* and *PpSPS3* had 13 exons and 12 introns, while *PpSPS4* had 14 exons and 13 introns (Figure 4B). The results of the *PpSPS* promoter analysis showed that there were 20 types of binding sites in all *PpSPS* (Figure 4C).

### 3.5. ALA-Regulated PpSPS-Related Genes

**Figure 5:**
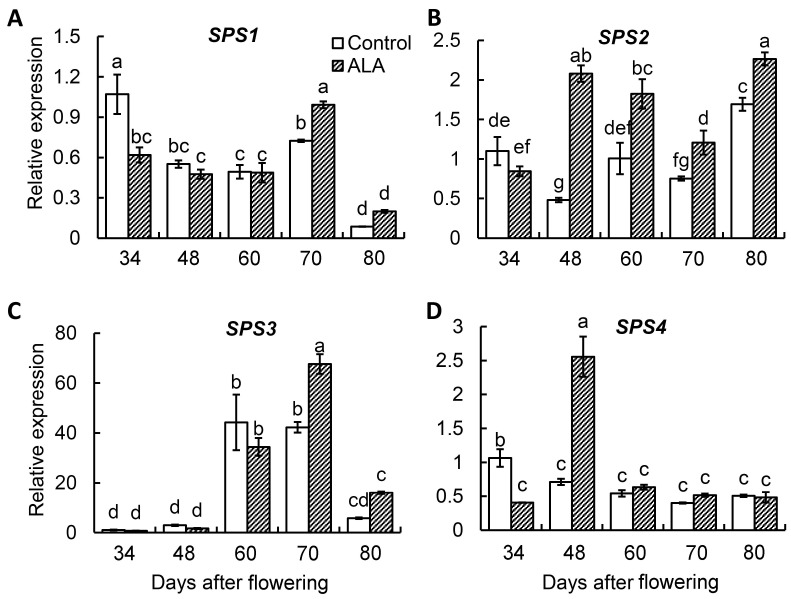
*PpSPS* gene expression in peach fruit after ALA treatment. (**A**): *SPS1* relative expression. (**B**): *SPS2* relative expression. (**C**): *SPS3* relative expression. (**D**): *SPS3* relative expression. The values are the means ± SE from three biological replicates. The same letters above the bars in each enzyme indicate no significant differences at *p* = 0.05 level.

The results of qRT-PCR showed that the expression of *PpSPS2* was significantly increased after 34 days after the flowering of ALA treatment (Figure 5B), while the expression of *PpSPS3* was significantly increased after 70 days after the flowering of ALA treatment (Figure 5C), and the expressions of *PpSPS1* and *PpSPS4* were not responsive to ALA (Figure 5A,D). Correlation analysis showed that *PpSPS2* gene expression was positively correlated with sucrose accumulation and SPS activity in peach fruit (Table S2 and Figure S1).

### 3.6. Subcellular Localization of PpSPS2 and PpSPS3

**Figure 6:**
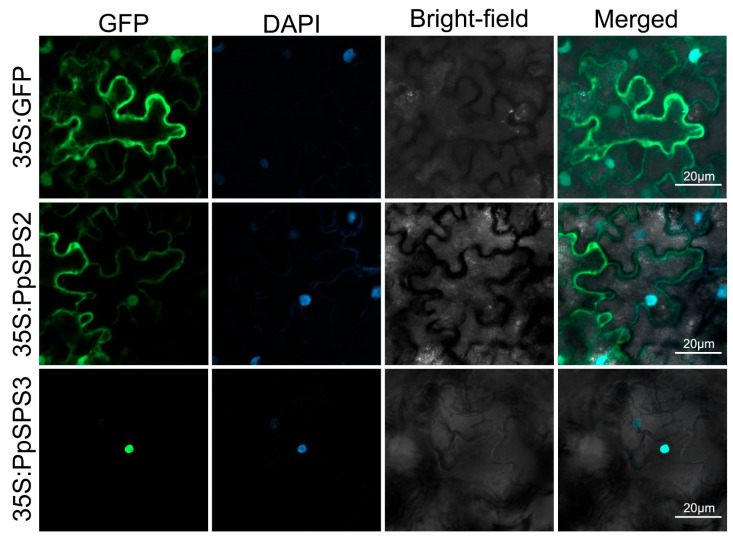
Subcellular localization of PpSPS2 and PpSPS3. GFP: green fluorescent protein. DAPI: 4′, 6-diamidino-2-phenylindole, a nuclear dye.

DAPI is a fluorescent dye that binds to most of the A and T bases in DNA. When DAPI is combined with double-stranded DNA, the maximum absorption wavelength is 358 nm, and the maximum emission wavelength is 461 nm. The emission light of DAPI is blue, and the emission wavelengths of DAPI and GFP overlap slightly, so it is possible to use this property for multiple fluorescence staining on a single sample. After staining, the green fluorescence and nuclear localization signals were observed under ultra-high resolution laser confocal microscopy (LSM800, Zeiss, Oberkochen, Germany). When the DAPI signal overlaps with the GFP signal, it indicates localization in the nucleus. The results of tobacco subcellular localization showed that PpSPS2 was mainly distributed in the cytoplasm and nucleus, while PpSPS3 was mainly distributed in the nucleus (Figure 6).

## 4. Discussion

Sucrose was found in many organ tissues, such as roots, stems, leaves, flowers and fruits, which indicates that SPS is a multifunctional protein. Studies have shown that SPS participated in and regulated photosynthetic carbon allocation in plants [28,29,30], which affected the partitioning between sucrose and starch [7], seed and pollen germination [31,32], cell differentiation, fiber cell wall synthesis [33], sugar accumulation and ripening of fruit [34]. In addition, it was also in response to abiotic stress, such as osmosis [35], low temperature [36], moisture [37] and drought [38].

Significant differences in SPS activity were found in different plants or at different growth and development stages of the same plant, suggesting the mechanism of regulating SPS activity is also relatively complex. In plants, SPS activity is regulated by their own development and surrounding environment. In peach fruit, the *SPS* expression in mature peaches was significantly higher than that in immature fruit [4]. SPS responded to various environmental and endogenous signal changes through phosphorylation and dephosphorylation [39]. Studies have shown that at least one PP2A complex protein phosphatase could promote the phosphating of SPS [7,39,40], while there are multiple kinases that could induce SPS phosphorylation [41]. In addition to responding to the abiotic stress mentioned earlier, plant SPS activity was regulated by light, which promoted SPS activity, while darkness did the opposite [42].

In addition, the activity of SPS was also regulated by its inhibitors and activators, including phosphate and sucrose-6-phosphate, and activators fructose-6-phosphate and 1,5-anhydroglucolol-6-phosphate. Although activators and inhibitors of these SPS have been discovered, further research on the activity of other small molecules on SPS is needed. Finally, interaction with proteins also affected SPS activity. There are regions in the plant SPS sequence that bind 14-3-3 proteins, which significantly inhibit SPS activity after binding to SPS [39]. There were few studies on the regulation of SPS activity by protein interaction, which is a scientific problem worthy of further study. In our study, ALA significantly improved PpSPS activity (Figure 2B) and increased the expression of *PpSPS2* in the peach fruits (Figure 4B). However, how ALA regulates PpSPS activity warrants further investigation.

Sucrose plays an important role in the transport of carbohydrates and their storage in plants [8]. We found ALA could significantly improve the sucrose of the peach fruits (Figure 2A). In plants, the *SPS* gene family usually consists of three subfamilies, namely A, B and C [43], and the expression patterns and amounts of different types of *SPS* subfamily genes in different tissues and organs of different plants are also not the same. This also suggested that different *SPS* genes were functionally differentiated [35,44,45]. In our study, we found that the *PpSPS* family consisted of four members: *PpSPS1*, *PpSPS2*, *PpSPS3* and *PpSPS4* (Table 2 and Figure 3).

Clearly, elucidating the interactions between different subunits of *SPS* and their relationship to SPS activity is of great scientific significance for revealing the role of SPS in sucrose metabolism. The results of tobacco subcellular localization showed that PpSPS2 was mainly distributed in the cytoplasm and nucleus, while PpSPS3 was mainly distributed in the nucleus (Figure 6). Thus, screening the transcription factors or key enzymes that interacted with PpSPS was an urgent task. In leaves, increased SPS activity led to an increase in sucrose content and a decrease in starch content [7]. However, during fruit development, SPS activity and sucrose content are controversial. In most cases, *SPS* expression and its activity are positively correlated with sucrose accumulation, but there is also a negative correlation [7,8,46]. Studies have shown that *MdSPSA2.3* was positively correlated with sucrose content during the development of apple fruits, and silencing of *MdSPSA2.3* significantly reduced sucrose accumulation in apple fruits [46]. In contrast, during the maturation of peach fruit, SPS activity was always low and negatively correlated with sucrose accumulation [45].

In summary, most of the current functional studies of SPS still focus on the determination of physiological indicators such as enzyme activity changes, while there are few studies at the biochemical and molecular level, and the specific gene function and molecular genetic mechanism of *SPS* are not clear. Plant SPS is a large molecular weight enzyme that contains other sequences in addition to the catalytic domain, which indicates that plant SPS must interact with other proteins to regulate their own activity, which is poorly studied. Although studies have shown that at least one PP2A complex protein phosphatase could promote the phosphating of SPS. However, this hypothesis has not yet been tested.

## Figures and Tables

**Table 1:** Effects of exogenous ALA treatment at different stages of fruit development on the quality of ‘Zhongyoutao 4’ nectarine.

Treatment	Fruit Weight (g)	Soluble Solid (%)	Soluble Sugar (%)	Titratable Acid (‰)	Soluble Protein (mg/g)	Vitamin C (mg/100 g)
Control	86.89 ± 2.26 b	15.85 ± 0.29 b	10.18 ± 0.30 b	3.48 ± 0.04 a	6.03 ± 0.18b	4.20 ± 0.31ab
ALA	98.79 ± 3.01 a	17.06 ± 0.45 a	11.64 ± 0.13 a	3.29 ± 0.04 b	7.25 ± 0.24 a	4.67 ± 0.16 a

Note: The same letter in a column indicates no significant difference at *p* = 0.05 level. The data on fruit weight, soluble solids and firmness are the means ± standard error of twenty biological replicates. Other data are the means ± standard error of four biological replicates.

**Table 2:** Detailed information on the *SPS* gene families in *Prunus persica*.

Gene Name	Gene ID	Genome Location	CDS Length (bp)	Protein Length (aa)	Mw (KDa)	Subcellular Localization
*PpSPS1*	Prupe.7G249900	Chr07: 21151882-21157785 (−)	3174	1057	118.17	Cytoplasmic
*PpSPS2*	Prupe.1G159700	Chr01:12702147-12709381 (−)	3207	1068	119.69	Cytoplasmic; Nuclear
*PpSPS3*	Prupe.1G483200	Chr01:40288494-40295210 (−)	3180	1059	118.26	Nuclear
*PpSPS4*	Prupe.8G003700	Chr08: 302873-308259 (−)	3078	1025	115.41	Cytoplasmic

## Data Availability

The data underlying this article are available in this paper. The datasets in this article were derived from sources in the public domain: National Center for biotechnology information (National Center for Biotechnology Information (nih.gov)) and the *Arabidopsis* information Resource (https://www.arabidopsis.org).

## Supplementary Materials

The following supporting information can be downloaded at: https://www.mdpi.com/article/10.3390/cimb46080469/s1, Table S1. Primers used in all the experiments. Table S2. Correlation analysis among sucrose content, PpSPS activity and *PpSPS* gene expression. Figure S1. Correlation and regression analysis among sucrose content, PpSPS activity and *PpSPS* gene expression.
