# Supplementary material for: ALA Promotes Sucrose Accumulation in Early Peach Fruit by Regulating SPS Activity
Source: Curr Issues Mol Biol. 2024 Jul 24;46(8):7944–54. doi: 10.3390/cimb46080469 (PMC11352516; doi:10.3390/cimb46080469)
Supplement: Supplementary file 1 [file cimb-46-00469-s001.zip › Figure S1.pdf]

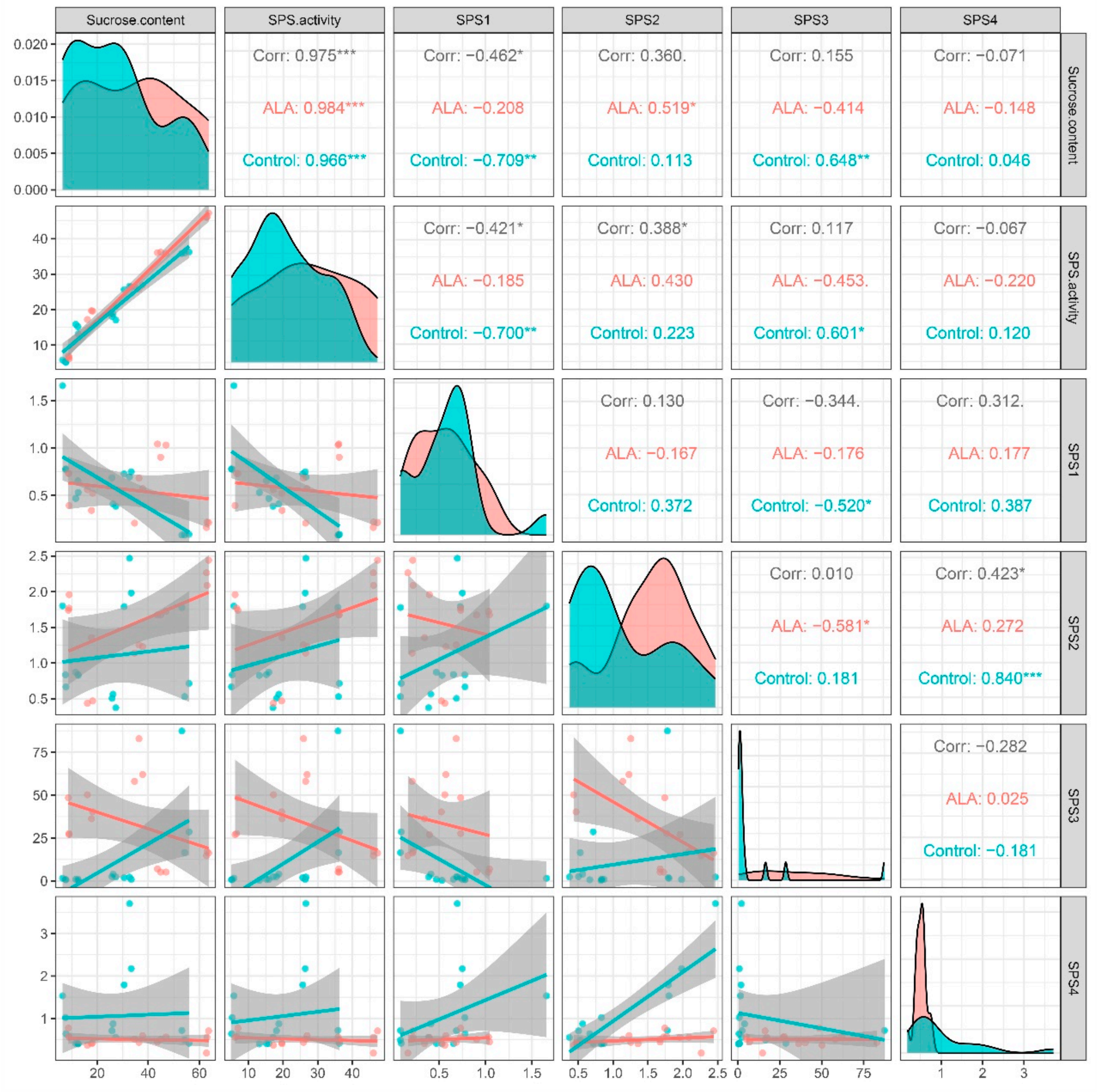

Figure S1. Correlation and regression analysis among sucrose content, PpSPS activity and *PpSPS* genes expression. Gray represents the correlation between the two indicators calculated, ignoring different treatments. The red color represents the correlation between the two indicators under ALA treatment. Green represents the correlation between the two indicators under the control. '*' represents significant at the level of 5%, $p<0.05$; '**' represents significant at the 1% level, $p<0.01$; '***' represents a significant level of 0.1%, $p<0.001$.
